# Role of poly(ADP-ribosyl)ation in a ‘two-hit’ model of hypoxia and oxidative stress in human A549 epithelial cells *in vitro*

**DOI:** 10.3892/ijmm.2013.1397

**Published:** 2013-05-29

**Authors:** KATALIN ERDÉLYI, PÁL PACHER, LÁSZLÓ VIRÁG, CSABA SZABÓ

**Affiliations:** 1 Department of Anesthesiology, The University of Texas Medical Branch, Galveston, TX 77555-1102, USA; 2 Section on Oxidative Stress and Tissue Injury, Laboratory of Physiologic Studies, National Institutes of Health/NIAAA, Bethesda, MD 20892-9413, USA; 3 Department of Medical Chemistry, Medical and Health Science Center, University of Debrecen, Debrecen H-4010, Hungary, USA; 4 Shriners Hospital for Children Galveston, Galveston, TX 77550-2725, USA

**Keywords:** hypoxia, oxidative stress, lung, poly(ADP-ribose) polymerase, epithelial cell, glutathione, DNA damage

## Abstract

A preceding hypoxic insult can sensitize the cells or the organism to a subsequent, second insult. The aim of the present study was to investigate the molecular mechanism of this phenomenon (often termed ‘two-hit’ injury paradigm), in an *in vitro* model of hypoxia/oxidative stress injury in A549 epithelial cells, with special emphasis on the role of the nuclear enzyme poly(ADP-ribose) polymerase-1 (PARP-1) in the process. Pre-exposure of the cells to 24 h hypoxia significantly reduced intracellular glutathione (GSH) levels, reduced mitochondrial activity and adenosine triphosphate (ATP) levels. However pre-exposure to hypoxia failed to induce any change in PARP-1 expression and activation, DNA single-strand breaks or plasma membrane integrity. Pre-exposure to hypoxia markedly increased the sensitivity of the cells to subsequent oxidative stress-induced DNA damage. Hydrogen peroxide (H_2_O_2_) induced a concentration-dependent increase in DNA breakage, PARP activation, depletion of intracellular ATP, inhibition of mitochondrial activity and two distinct parameters that quantify the breakdown of plasma membrane integrity (propidium iodide uptake or lactate dehydrogenase release). PARP-1 activation played a significant role in the H_2_O_2_-induced cell death response because PARP activation, depletion of intracellular ATP, inhibition of mitochondrial activity, and the breakdown of plasma membrane integrity were attenuated in cells with permanently silenced PARP-1. Based on measurement of the endogenous antioxidant GSH, we hypothesized that the mechanism of hypoxia-mediated enhancement of H_2_O_2_ involves depletion of the GSH during the hypoxic period, which renders the cells more sensitive to a subsequent DNA single-strand break elicited by H_2_O_2_. DNA strand breakage then activates PARP-1, leading to the inhibition of mitochondrial function, depletion of ATP and cell necrosis. PARP-1 deficiency protects against the cytotoxicity, to a lesser degree, by protecting against GSH depletion during the hypoxic period, and, to a larger degree, by maintaining mitochondrial function and preserving intracellular ATP levels during the subsequent oxidative stress period.

## Introduction

The molecular mechanisms underlying the various ‘two-hit models’ of injury have been subject to intensive investigations in various models of critical illness (1–7). While a slight degree/shorter period or repeated short periods of hypoxia can protect cells or tissues from a subsequent, more severe form of injury (‘ischemic preconditioning’), more severe degrees of pre-existing injuries can make the organism ultrasensitive to a second insult in the paradigm of various forms of ‘two-hit models’.

In the present study, we established an *in vitro* model of two-hit injury involving pre-exposure to hypoxia, followed by a second challenge induced by oxidative stress in cultured human lung epithelial cells. Due to the role of the nuclear enzyme poly(ADP-ribose) polymerase-1 (PARP-1) in the pathogenesis of various diseases associated with oxidative or nitrative stress (8–10), we also investigated the potential contribution of the activation of PARP-1 (the major PARP isoform) to the cell injury associated with the ‘two-hit’ response.

## Materials and methods

### Reagents

Unless specified otherwise, all the reagents were purchased from Sigma-Aldrich Co. (St. Louis, MO, USA).

### Cell culture

The A549 human lung adenocarcinoma cell line was grown in RPMI-1640 medium containing 10% fetal bovine serum (FBS; PAA Laboratories, Dartmouth, MA, USA) 100 U/ml penicillin and 100 μg/ml streptomycin (Invitrogen, Carlsbad, CA, USA) at 37°C, 5% CO_2_. Stable gene silencing of PARP-1 with lentiviral infection (yielding shPARP-1 cells) was performed as previously described (11), i.e., control cells were subjected to an identical procedure except that they were transfected with a non-coding silencing vector (11).

### In vitro model of hypoxia

Cell culture plates were placed in gas-tight incubation chambers (Billups-Rothenberg Inc., Del Mar, CA, USA) and the chamber atmosphere was replaced by flushing the chamber with 95% N_2_/5% CO_2_ mixture at a flow rate of 25 l/min for 5 min. Hypoxia was maintained by clamping and the chambers were incubated for 24 h at 37°C, as described in a previous study (12). Following hypoxia, cells were incubated for the indicated period at 37°C in a 5% CO_2_ atmosphere in the presence or absence of various concentrations of hydrogen peroxide.

### Measurement of cellular glutathione (GSH) content

Total cellular GSH content was measured using the OxiSelect™ Total Glutathione (GSSG/GSH) assay kit (Cell Biolabs, Inc., San Diego, CA, USA), as previously described (13). This kit provided the enzyme glutathione reductase, which reduced oxidized glutathione (GSSG) to reduced GSH in the presence of NADPH. Subsequently, a chromogen reacted with the thiol group of GSH to produce a colored compound that was absorbed at 405 nm. The rate of chromophore production was proportional to the concentration of GSH within the sample. The rate was determined from the absorbance change over time.

### Single-cell electrophoresis (comet assay)

Broken DNA was allowed to unwind under alkaline conditions and formed comet-like structures after cell lysis and electrophoresis. Following trypsinization, single-cell suspension was treated with hydrogen peroxide for 10 min. Single-stranded DNA breaks were assayed by comet assay using a CometAssay kit (Trevigen, Gaithersburg, MA, USA), as previously described (14). Single cells were exposed to an electric field in agarose gel and stained with SYBR-Green. Labeled DNA was then visualized under fluorescence microscopy.

### Measurement of intracellular adenosine triphosphate (ATP) levels

To measure intracellular ATP levels, a CellTiter-Glo Luminescent Cell Viability assay (Promega, Madison, WI, USA) based on ATP requiring luciferin-oxyluciferin conversion mediated by a thermostable luciferase generating a stable ‘glow-type’ luminescent signal was used. The cells were lysed in 100 μl of CellTiter-Glo reagent according to the manufacturer’s instructions and the luminescent signal was recorded for 1 sec on a high sensitivity luminometer (Synergy 2, Biotek, Winooski, VT, USA) (15). Changes in ATP concentration were calculated as a percentage of the untreated control.

### Western blot analysis

Western blot analysis for poly(ADP-ribose) (PAR) polymers was conducted as previously described (11). Cells were washed once with phosphate buffered-saline (PBS) and collected by scraping into 200 μl ice-cold lysis buffer containing 62.5 mM Tris-HCl (pH 6.8), 2% SDS, 10% glycerol, 1 mM PMSF, and protease inhibitors. The extracts were sonicated, and the supernatants were collected following centrifugation. The protein concentration was determined by BCA Protein assay (Thermo Scientific, Rockford, IL, USA). Prior to boiling, samples were digested with 50 mM dithiothreitol (DTT) and 0.1% bromophenol blue. Protein (20 μg) was loaded onto 8% polyacrylamide gels. Proteins were separated by electrophoresis and then transferred to nitrocellulose membrane. For immunoblotting, membranes were blocked with 5% non-fat milk in Tris-buffered saline (TBS) for 60 min. Primary antibody against poly(ADP-ribose) polymers (Trevigen, Gaithersburg, MA, USA) or against PARP-1 (Cell Signaling Technology, Inc., Danvers, MA, USA) were applied at 1,000-fold of dilution in blocking buffer, overnight at 4°C. After washing 3 times in TBS containing 0.2% Tween-20 (TBST), secondary antibody (peroxidase-conjugated goat anti-rabbit) and peroxidase-conjugated anti-actin (Santa Cruz Biotechnology, Inc., Santa Cruz, CA, USA) were applied at 4,000-fold of dilution in blocking buffer for 1 h at room temperature. Blots were washed 3 times in TBST, once in TBS and were then incubated in enhanced chemiluminescence substrate and Supersignal West Pico Chemiluminescent substrate (Thermo Scientific), and exposed to photographic film.

### MTT mitochondrial activity assay

The MTT assay was performed as previously described (16). Briefly, cells were treated after normoxia or hypoxia with hydrogen peroxide in 96-well plates. Then, 24 h later 3-(4,5-dimethyl-2-thiazolyl)-2,5-diphenyl-2H-tetrazolium bromide (MTT, Calbiochem, EMD BioSciences, San Diego, CA, USA) was added to the cells (0.5 mg/ml) for an additional hour. The medium was then aspirated and the formazan crystals were dissolved by the addition of 100 μl isopropanol. Optical density was detected on a Synergy 2 reader (BioTek Instruments, Inc., Winooski, VT, USA) at 570 nm with background measurement at 690 nm. Results are shown as percentage compared to the untreated control.

### Lactate dehydrogenase (LDH) assay

LDH release was measured as previously described (17). Briefly, cell culture supernatant (30 μl) was mixed with 100 μl freshly prepared LDH assay reagent and the changes in absorbance were read kinetically. LDH release values are shown as V_max_ (mOD/min).

### Measurement of plasma membrane integrity

Plasma membrane integrity was measured by propidium iodide (PI) uptake as described in a previous study (18). Briefly, the cells were stained with 5 μg/ml PI for 15 min. Detached and trypsinized cells were then collected, washed once with PBS and analyzed by the Guava Easycyte Plus flow cytometry system (Millipore, Billerica, MA, USA).

### Experimental protocols

**Figure 1:**
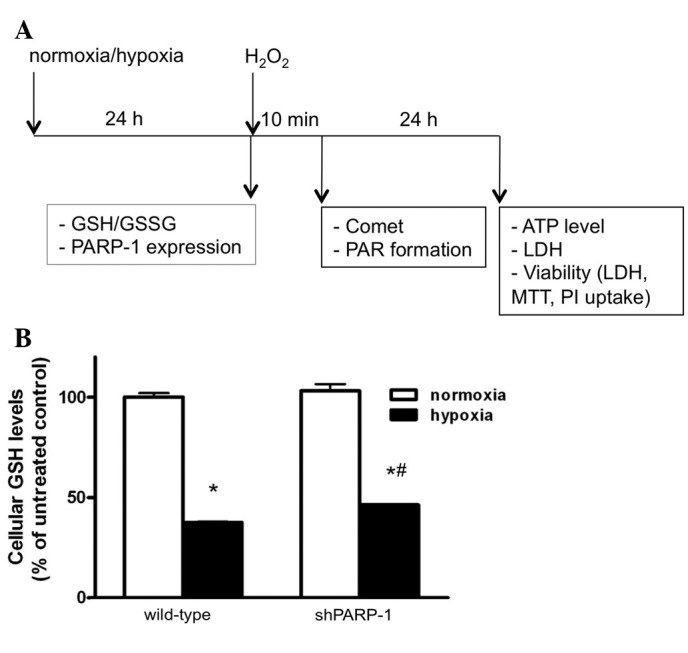
(A) Experimental design: an *in vitro* model of ‘two-hit’ injury in cultured A549 human lung epithelial cells. Control and shPARP-1 cells were grown to form an 80% confluent monolayer and then subjected to either hypoxia or normoxia for 24 h. Immediately after hypoxia, the cellular glutathione (GSH) content and poly(ADP-ribose) polymerase-1 (PARP-1) protein level was measured or cells were treated with H_2_O_2_ for 10 min, followed by measuring of the poly(ADP-ribose) polymer (PAR) formation by western blotting or the sensitivity to single-strand DNA breakage by comet assay. The prolonged effect of oxidative stress on cells undergoing hypoxia was examined by cell viability and toxicity assays at 24 h after H_2_O_2_ exposure. (B) Effect of hypoxia on intracellular GSH levels in cultured A549 human lung epithelial cells. Cells were incubated in normoxia or in a hypoxic chamber for 24 h, followed by measurement of cellular GSH content. A significant reduction of cellular GSH content was measured in both the control and shPARP-1 cells. Data are shown as mean ± SEM (n=6). ^*^P<0.05 shows a significant difference in the response of cells in hypoxia compared to normoxia; ^#^P<0.05 shows a significant difference between wild-type vs. shPARP-1 cells.

Control cells and cells with stably silenced PARP-1 (shPARP-1) were exposed to 24 h of hypoxia followed by the determination of cellular GSH levels, as well as quantification of PARP-1 expression (by western blotting). At 24 h, the cells were subjected to oxidative stress with hydrogen peroxide. Ten minutes later, DNA damage was assessed by the comet assay and PARP activation was assessed by western blotting for PAR polymers. At 24 h after the H_2_O_2_ challenge, cell viability was detected by the MTT and LDH assays, and by PI uptake, then cellular ATP levels were quantified (Fig. 1A).

### Statistical analysis

Data were shown as the means ± SEM. One-way ANOVA was applied for statistical analysis, while the Tukey’s post hoc test was used for the determination of significance. P<0.0.5 was considered statistically significant. Statistical calculations were performed using Graphpad Prism 5 analysis software. Experiments were performed at least 3 times on different days.

## Results

### Hypoxia reduced the cellular GSH content without affecting the expression of PARP-1 protein

**Figure 2:**
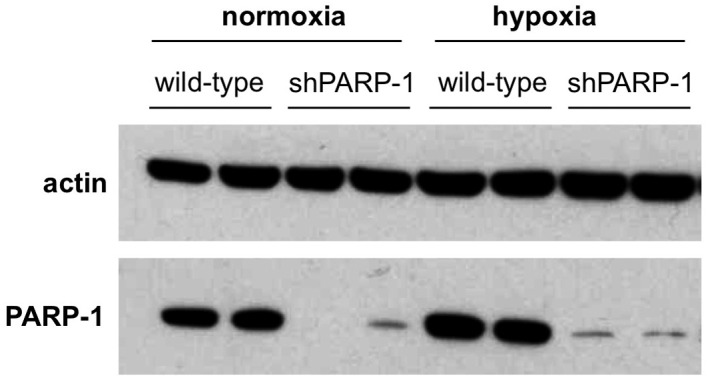
Effect of 24 h of hypoxia on poly(ADP-ribose) polymerase-1 (PARP-1) protein expression in cultured A549 human lung epithelial cells. Following 24 h of hypoxia, PARP-1 content was determined by western blotting. Actin was used as an invariant control. The protein level of PARP-1 was not affected in the control cells after 24 h of hypoxia. shPARP-1 cells exhibited markedly reduced levels of PARP-1. Representative western blots of determination are shown (n=3).

**Figure 3:**
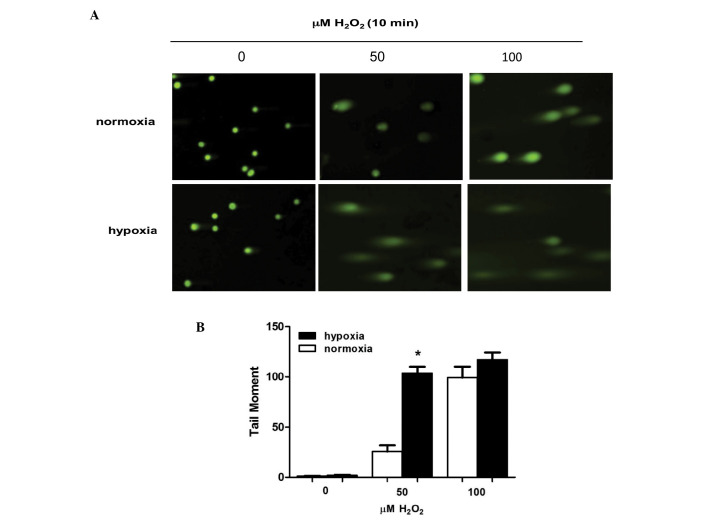
Effect of pre-exposure to hypoxia on the sensitivity of cultured A549 human lung epithelial cells to exhibit DNA single-strand break in response to H_2_O_2_. DNA single-strand break was measured with the comet assay. After 24 h of hypoxia or normoxia, wild-type cells were subjected to 50 or 100 μM H_2_O_2_ for 10 min. Single cells were then exposed to an electric field in agarose gel and stained with SYBR-Green. Labeled DNA was visualized under fluorescence microscopy. (A) Representative images. (B) Main tail moments as an index of DNA damage. Hypoxia rendered the cells significantly more sensitive to 50 μM H_2_O_2_-induced single-strand DNA breakage. Data are shown as mean ± SEM (n=6). ^*^P<0.05 shows a significant difference in the response of cells in hypoxia compared to normoxia.

**Figure 4:**
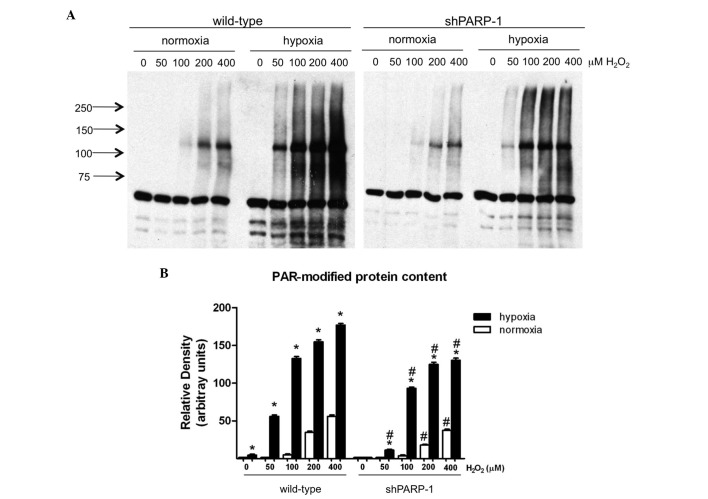
Effect of pre-exposure to hypoxia on the sensitivity of cultured A549 human lung epithelial cells to exhibit poly(ADP-ribose) polymer (PAR) formation in response to H_2_O_2_. H_2_O_2_-induced PAR formation in control and shPARP-1 cells either in normoxia or in 24 h hypoxia was measured by immunoblotting. After 24 h of hypoxia or normoxia, the cells were treated with increasing concentrations of H_2_O_2_ for 10 min. The cells that were pre-exposed to hypoxia exhibited significantly higher amounts of PAR. Reduced PAR synthesis was measured in shPARP-1 cells, compared to wild-type cells. (A) Representative western blot analysis. (B) Results evaluated by densitometry and analyzed statistically. In part A, mean ± SEM values of n=3 are shown. ^*^P<0.05 shows significant difference in the response of cells in hypoxia compared to normoxia; ^#^P<0.05 shows a significant difference between wild-type vs. shPARP-1 cells.

**Figure 5:**
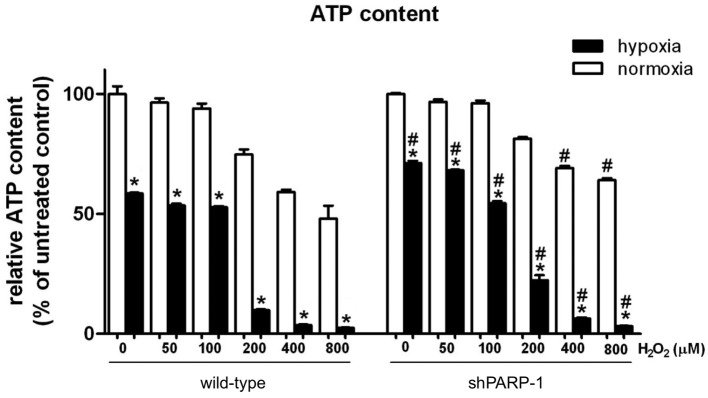
Effect of pre-exposure to hypoxia on the H_2_O_2_-induced changes in cellular adenosine triphosphate (ATP) content in cultured A549 human lung epithelial cells. After 24 h of hypoxia or normoxia, the cells were treated with increasing concentrations of H_2_O_2_ for 24 h. Oxidative stress decreased the cellular ATP content in wild-type and shPARP-1 cells. Cells that were pre-exposed to hypoxia exhibited significantly higher decreases in ATP level after H_2_O_2_, compared to cells that were not pre-exposed to hypoxia. shPARP-1 cells were significantly protected against an H_2_O_2_-induced decrease in ATP, compared to wild-type cells. Data are shown as mean ± SEM (n=3). ^*^P<0.05 shows a significant difference in the response of cells in hypoxia compared to normoxia; ^#^P<0.05 shows significant difference between wild-type vs. shPARP-1 cells.

**Figure 6:**
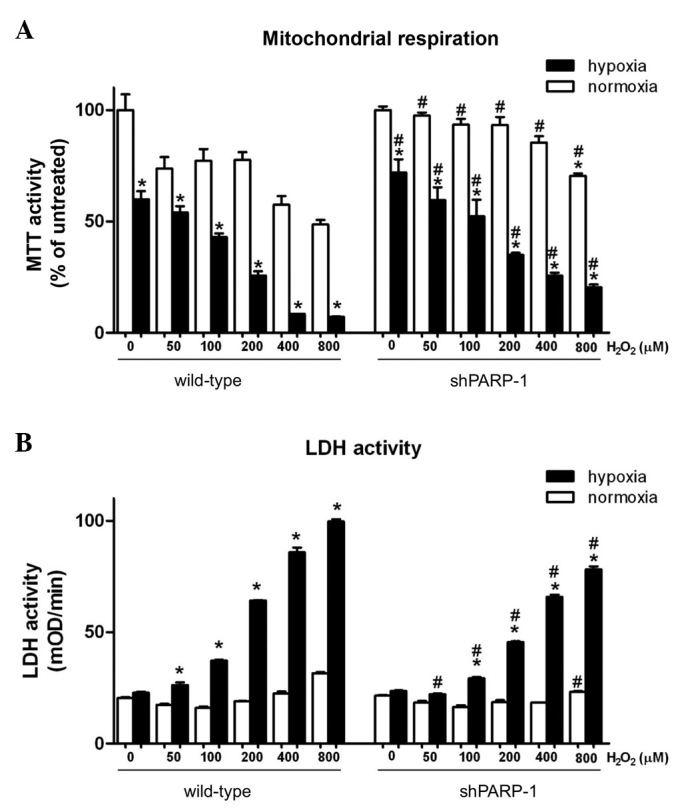
Effect of pre-exposure to hypoxia on the H_2_O_2_-induced changes in (A) mitochondrial MTT conversion and (B) cell viability in cultured A549 human lung epithelial cells. (A) Mitochondrial MTT conversion and (B) decreases in cell viability were evaluated by the measurement of lactate dehydrogenase (LDH) release into the cell culture supernatant. After 24 h of hypoxia or normoxia, the cells were treated with increasing concentrations of H_2_O_2_ for 24 h. Oxidative stress decreased the MTT activity and increased LDH release in wild-type and shPARP-1 cells. Cells that were pre-exposed to hypoxia exhibited significantly more pronounced responses to H_2_O_2_, compared to cells that were not pre-exposed to hypoxia. shPARP-1 cells were significantly protected against H_2_O_2_-induced alterations, compared to wild-type cells. Data are shown as mean ± SEM (n=3). ^*^P<0.05 shows a significant difference in the cell response in hypoxia compared to normoxia. ^#^P<0.05 shows a significant difference between wild-type and shPARP-1 cells.

**Figure 7:**
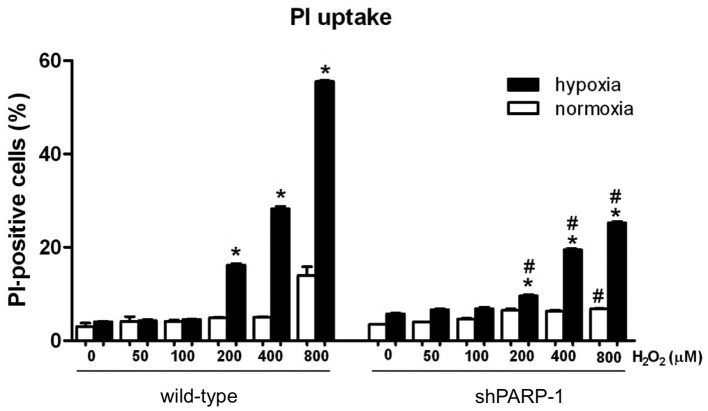
Effect of pre-exposure to hypoxia on the H_2_O_2_-induced plasma membrane injury in cultured A549 human lung epithelial cells. Plasma membrane injury was assessed by propidium iodide (PI) staining. After 24 h of hypoxia or normoxia, the cells were treated with increasing concentrations of H_2_O_2_ for 24 h. Oxidative stress induced plasma membrane injury in wild-type and shPARP-1 cells. Cells that were pre-exposed to hypoxia exhibited significantly more pronounced responses to H_2_O_2_, compared to cells that were not pre-exposed to hypoxia. shPARP-1 cells were significantly protected against H_2_O_2_-induced alterations, compared to wild-type cells. Data are shown as mean ± SEM (n=3). ^*^P<0.05 shows a significant difference in the cell response in hypoxia compared to normoxia; ^#^P<0.05 shows a significant difference between wild-type and shPARP-1 cells.

Hypoxia significantly reduced the intracellular GSH levels to 37±2% of normoxic values in wild-type cells and to a smaller degree (to 46±2% of normoxic values) in the shPARP-1 cells (Fig. 1B). The expression level of PARP-1 remained stable after hypoxia. As expected, shPARP-1 cell lines exhibited an efficient reduction of the corresponding protein, with a small amount of residual PARP-1 enzyme remaining to be identified (Fig. 2). Hypoxia, by itself, did not induce an increase in DNA strand breakage (Fig. 3) and did not activate PARP-1 (Fig. 4). Although these findings suggest that the current protocol of hypoxia used, by itself, does not result in a significant degree of PARP-1 activation (or PARP-mediated decreases in cell viability), cell viability was reduced by hypoxia in a partially PARP-1-dependent manner. For instance, when measured at 48 h after the start of the hypoxia (i.e., 24 h of hypoxia and 24 h of reoxygenation), ATP levels were reduced to 58±2% of control in the wild-type cells, while these levels were maintained at 71±3% of control in the shPARP-1 cells (Fig. 5). Mitochondrial activity, as evaluated by the MTT assay, showed a similar pattern (60±7% of control in the wild-type cells, and 71±10% of control in the shPARP-1 cells) (Fig. 6). However, hypoxia alone did not create a significant loss of cell membrane integrity as there were no increases in LDH levels in the supernatant, nor did hypoxia induce any detectable increase in the percentage of PI-positive cells (Fig. 7).

### Pre-exposure to hypoxia increased sensitivity of cells to subsequent, oxidative stress-induced DNA damage

H_2_O_2_ (50 or 100 μM) induced a concentration-dependent increase in DNA breakage, as assessed by single-cell electrophoresis (comet assay) (Fig. 3A). Cells that were pre-exposed to hypoxia exhibited higher sensitivity to the subsequent oxidative stress challenge, as evidenced by the significantly higher tail moment developing in response to the lower dose of H_2_O_2_ used (Fig. 3B). In accordance with the fact that DNA strand breakage is a canonical trigger of PARP activation (8–10), H_2_O_2_ induced a concentration-dependent increase in the poly(ADP-ribose) polymer (PAR) level in all cell groups. This was evidenced as a smear of 115–150 kDa, indicating auto-modified PARP-1 (11). While in cells that previously underwent hypoxia, baseline DNA strand breakage or PARP activation remained unchanged, H_2_O_2_ treatment triggered a substantially more pronounced PARP-1 activation response, i.e., cells contained more positive bands with the most immunopositivity identified in the region of 75–150 kDa (Fig. 4). In shPARP-1 cells H_2_O_2_ challenge only resulted in a slight elevation of cellular PAR polymer content. However, the H_2_O_2_-induced PARylation response was more elevated in cells that were pre-exposed to hypoxia (Fig. 4). Despite the fact that shPARP-1 cells contained substantially lower amounts of PARP-1 compared to wild-type cells, when these cells were previously exposed to hypoxia, the H_2_O_2_-induced PARylation response was higher than the corresponding response in wild-type cells that were previously not exposed to hypoxia.

### Role of PARP-1 in cell death induced H_2_O_2_ with or without pre-exposure to hypoxia

H_2_O_2_ caused a similar degree of concentration-dependent decrease in the ATP content of both the control and shPARP-1 cells (Fig. 5). When the cells were pre-exposed to hypoxia, this H_2_O_2_-induced drop in ATP content was amplified, and PARP-1 deletion provided significant protection against this amplification (Fig. 5). Similar patterns were observed with respect to various indices of cell viability: the H_2_O_2_-induced decreases in MTT activity, LDH release into the supernatant, and increases in the proportion of PI-positive cells, were all more pronounced in the cells that were previously subjected to hypoxia, than the corresponding control cells, and this hypersensitivity was attenuated in the shPARP-1 cells, compared to wild-type cells (Figs. 6 and 7). The control cells (not pre-exposed to hypoxia) tolerated the H_2_O_2_ exposure relatively well: even at the highest concentration (800 μM) of H_2_O_2_ used, there was only a <50% decrease in cellular ATP content and mitochondrial MTT conversion, with the two responses being ameliorated by PARP-1 deficiency (Figs. 5 and 6). Moreover, the control cells (not pre-exposed to hypoxia), did not exhibit any sign of membrane integrity breakdown after H_2_O_2_ exposure, with the exception of the 800 μM concentration, where a slight, PARP-1-dependent increase in PI positivity and LDH release were observed (Fig. 7).

## Discussion

The principal finding of the current study is that a 24 h pre-exposure of human lung epithelial cells to hypoxia renders them hypersensitive to subsequent oxidative stress in a partially PARP-1-dependent manner. The cells that were not pre-exposed to hypoxia tolerated the H_2_O_2_ exposure relatively well across the entire concentration range used, with a decrease in ATP and a slight increase in PI positivity noted at the highest concentration of H_2_O_2_ (800 μM).

Similarly, hypoxic exposure by itself did not produce cytotoxicity (as evidenced by lack of LDH release or lack of change in PI positivity), although it created a partial energetic imbalance (evidenced by a reduced degree of mitochondria-dependent MTT conversion), and a decrease in the intracellular ATP levels. The hypoxia-preexposed cells responded to a markedly more pronounced cell death response following exposure to H_2_O_2_ compared to the control cells (i.e., cells not pre-exposed to hypoxia). For instance, at 200 μM H_2_O_2_, ATP levels and mitochondrial MTT conversion were reduced by 75–80% of baseline values in the cells that were pre-exposed to hypoxia, while the same parameters were reduced by only 20–25% in cells that were not subjected to hypoxia. Thus, it can be determined that decreases in mitochondrial MTT conversion and cellular ATP content that are <50% (e.g., in response to 400–800 μM H_2_O_2_ in the non-hypoxic control group, or in response to 50–100 μM H_2_O_2_ in the cells that were pre-exposed to hypoxia) do not result in any detectable changes in membrane permeability (i.e., lack of increase in LDH release or PI positivity), while insults that reduce the ATP levels <50% are generally associated with a breakdown of cell membrane integrity in the current experimental system.

The functional importance of PARP-1 in the cytotoxic response in our experimental model is supported by the fact that shPARP-1 cells tolerated the hypoxic/oxidative challenge better compared to the wild-type cells, as evidenced by an improved maintenance of ATP, and a lower degree of suppression of mitochondrial activity, coupled with a lower degree of LDH release and lower proportion of PI-positive cells. The rank order of the degree of PARP activation also mirrored the degree of energetic and functional alterations across the four experimental groups studied: the most pronounced response was noted in the wild-type group subjected to hypoxia/H_2_O_2_, followed by the shPARP-1 group subjected to hypoxia/H_2_O_2_, followed by the wild-type group subjected to H_2_O_2_ without hypoxia, and finally (the least degree of PARP activation and the least degree of energetic and functional changes) the shPARP-1 group subjected to H_2_O_2_ without hypoxia (Figs. 5–7). The protective effect of PARP-1 deficiency, as well as the identical rank order for PARP-1 activation and the functional changes are all in strong support of the hypothesis that activation of PARP-1 plays a direct causal role in this process. According to the ‘canonical’ pathway of PARP-1 activation/cytotoxicity, DNA strand breakage activates PARP-1, which, in turn, triggers a marked depletion of its substrate, cellular NAD^+^, and, secondarily, cellular ATP, leading to mitochondrial dysfunction and cell death via the necrotic path (19–21). The cell responses elicited by H_2_O_2_ in this study are in concordance with those of that model. However, during the hypoxic period, it is likely that mechanisms other than activation of PARP-1 play a key role in the process of ATP depletion, because hypoxia alone did not induce a PARylation response. Of note, the current experimental design did not measure the full time-course of cellular ATP during the 24 h of hypoxia and the subsequent reoxygenation. However, in other studies utilizing 24 h hypoxia in epithelial cells, ATP levels were measured in more detail (15,22). Based on these studies, we hypothesized that in the current experiments hypoxia induces a marked decrease in cellular ATP levels by 24 h, while the levels measured at 48 h after the initiation of hypoxia (i.e., at 24 h hypoxia + 24 h subsequent to normoxia) result from a partial recovery of cellular ATP. Thus, at the time point of the H_2_O_2_ exposure in the current protocol (immediately after the end of the 24 h of hypoxia), the cells were subjected to oxidative stress at a time when intracellular ATP levels were the most severely depleted. In this respect, the current *in vitro* model represents a form of the ‘two-hit’ injury model where the second ‘hit’ (oxidative stress) follows, in short succession, the end of the first ‘hit’ (hypoxia). This type of response, is relatively common, because revascularization of ischemic organs *in vivo* is typically associated with an oxidative (and nitrative) stress ‘burst’ response.

Although the functionality of the epithelial cell monolayers was not assessed in the current experimental model, previous studies have demonstrated that PARP-1 activation is an important regulator of epithelial cell permeability both *in vitro* and *in vivo* in various models of critical illness (23–26). Therefore, it is likely that PARP-1-dependent alterations in epithelial cell energetics also translate to functional alterations in epithelial barrier function. Moreover, in addition to being a regulator of cellular bioenergetics (8,11,15,18–22), PARP-1, also affects pro-inflammatory gene transcription and inflammatory mediator production (27–32). Additional studies are needed to investigate whether the well-known propensity of the ‘two-hit’ models of critical illness to an enhanced inflammatory mediator production can also be reproduced in the current *in vitro* system, and whether PARP activation is important in these responses.

The precise early-stage trigger of PARP-1 overactivation (and subsequent cellular dysfunction) in the current experimental model was investigated. Based on our findings showing that H_2_O_2_ induces a more pronounced degree of DNA strand breaks in the cells pre-exposed to hypoxia than in the control cells (Fig. 3), we hypothesize that hypoxia renders the nuclear DNA more sensitive to oxidative stress, either by permitting the oxidants to penetrate the cells better, and/or by causing a more pronounced degree of secondary oxidant production (33), for instance by the promotion of mitochondrial dysfunction and mitochondrial oxidant production. In general, oxidative stress reflects an imbalance between the systematic occurrence of reactive oxygen species and the ability of the biological system to detoxify the harmful intermediates or to repair the resulting damage.

One of the key intracellular antioxidants is GSH, a key intracellular tripeptide thiol composed of glutamic acid, cysteine and glycine. GSH functions as an antioxidant, preventing damage to cell components caused by reactive oxygen species (34). It exists in reduced GSH and oxidized GSSG form. In the reduced state, the thiol group of the cysteine provides reduction equivalents to other unstable molecules, such as reactive oxygen species. After donating the electron, GSH becomes reactive, but reacts readily with another oxidized glutathione to form glutathione disulfide (GSSG). Under normal circumstances >90% of the total glutathione pool is in the reduced state (GSH), while the remaining glutathione pool is in the disulfide form (GSSG). The higher concentration of GSH is due to the glutathione reductase enzyme, which is constitutively active and inducible following oxidative stress (34). Earlier studies demonstrated that pharmacological depletion of intracellular GSH with L-buthionine-(S,R)-sulfoximine (an inhibitor of γ-glutamylcysteine synthetase) renders endothelial cells hypersensitive to oxidative and nitrative stress and PARP activation (35,36). Therefore, GSH levels were measured in our experiments and hypoxia was observed to result in a substantial decrease in its levels (Fig. 1). In accordance with previous studies using pharmacological PARP inhibitors such as 3-aminobenzamide (37), the hypoxia-mediated GSH depletion was, in the current experiments, at least partially dependent on the presence of PARP-1. GSH depletion has been previously shown to exacerbate the degree of oxidant-mediated DNA damage in a variety of experimental systems *in vitro*(38–40). Therefore, the above findings are consistent with the hypothesis that GSH depletion is a causative step in the process of hypoxia-associated, PARP-1-mediated enhancement of oxidative stress-induced cytotoxicity in the current, ‘two-hit’ *in vitro* experimental model. The present study also supports the view that restoration of endogenous antioxidants, such as GSH and/or pharmacological inhibition of PARP-1, can be of benefit in various models of critical illness, including ‘two-hit models’ and other pathophysiological conditions where hypoxia is followed by a subsequent oxidative stress-mediated insult.

## Abbreviations

ATP: adenosine triphosphate
GSH: glutathione
H_2_O_2_: hydrogen peroxide
MTT: 3-(4,5-dimethyl-2-thiazolyl)-2,5-diphenyl-2H-tetrazolium bromide
LDH: lactate dehydrogenase
PI: propidium iodide
PAR: poly(ADP-ribose) polymer
PARP: poly(ADP-ribose) polymerase
